# Lifespan and Stress Resistance in *Drosophila* with Overexpressed DNA Repair Genes

**DOI:** 10.1038/srep15299

**Published:** 2015-10-19

**Authors:** Mikhail Shaposhnikov, Ekaterina Proshkina, Lyubov Shilova, Alex Zhavoronkov, Alexey Moskalev

**Affiliations:** 1 Engelhardt Institute of Molecular Biology, Russian Academy of Sciences, Moscow, 119991, Russia; 2 Institute of Biology of Komi Science Center of Ural Branch of RAS, Syktyvkar, 167982, Russia; 3 Insilico Medicine, Inc, Johns Hopkins University, ETC, B301, Baltimore, MD, 21218, USA

## Abstract

DNA repair declines with age and correlates with longevity in many animal species. In this study, we investigated the effects of *GAL4*-induced overexpression of genes implicated in DNA repair on lifespan and resistance to stress factors in *Drosophila melanogaster.* Stress factors included hyperthermia, oxidative stress, and starvation. Overexpression was either constitutive or conditional and either ubiquitous or tissue-specific (nervous system). Overexpressed genes included those involved in recognition of DNA damage (homologs of *HUS1, CHK2*), nucleotide and base excision repair (homologs of *XPF, XPC* and AP-endonuclease-1), and repair of double-stranded DNA breaks (homologs of *BRCA2, XRCC3, KU80* and *WRNexo*). The overexpression of different DNA repair genes led to both positive and negative effects on lifespan and stress resistance. Effects were dependent on *GAL4* driver, stage of induction, sex, and role of the gene in the DNA repair process. While the constitutive/neuron-specific and conditional/ubiquitous overexpression of DNA repair genes negatively impacted lifespan and stress resistance, the constitutive/ubiquitous and conditional/neuron-specific overexpression of *Hus1, mnk, mei-9, mus210*, and *WRNexo* had beneficial effects. This study demonstrates for the first time the effects of overexpression of these DNA repair genes on both lifespan and stress resistance in *D. melanogaster*.

Aging is a multifactorial process caused by a wide range of physiological phenomena and changes in the functioning of different biological pathways^1^. Over the course of an organism’s lifespan, age-dependent mutations accumulate and are assumed to contribute to aging and age-related diseases^2^. While in some organisms, this may not the case^3^ in rodents and humans, many studies have shown that the level of DNA damage increases with age^2^. This damage includes abasic sites, DNA oxidation, DNA alkylation, DNA glycation, DNA cross-linkages, indigenous DNA adducts, and DNA strand breaks (Fig. 1).

The observed age-dependent increase in DNA damage is primarily linked to a decrease in the activity of various DNA repair processes^2^,^4^, such as base excision repair (BER), nucleotide excision repair (NER), mismatch repair (MMR), single-strand break (SSB) repair, double-strand break repair (DSBR) by homologous recombination (HR), single strand annealing (SSA), and non-homologous end joining (NHEJ) mechanisms (Supplementary Table S1). Thus, the increase in DNA damage is coupled with a simultaneous reduction in DNA repair, and this is accompanied by an accumulation of somatic mutations in model organisms such as fruit flies (*Drosophila melanogaster)*^5^, mice^6^, and humans^7^. The accumulation of mutations leads to carcinogenesis, higher numbers of unfit cells, and aging at the cellular, tissue, and organism levels^8^,^9^.

There is a positive relationship between organismal lifespan and efficiency of DNA damage repair. As shown in comparative studies performed on seven mammalian species, species longevity increases with the efficiency of DNA excision repair (ER)^10^. The enzymatic activity of poly (ADP-ribose) polymerase 1 (PARP1), a sensor of DNA strand breaks, positively correlates with maximum lifespan in 13 species of mammals^11^. Тhe level of Ku80, a DNA double strand break (DSB) recognition protein, in humans, cows, and mice is also strongly correlated with longevity^12^.

Species studied for long lifespan, such as naked mole rat *Heterocephalus glaber*, Brandt’s bat *Myotis brandtii*, and bowhead whale *Balaena mysticetus,* are characterized by higher numbers of copies or expression of genes controlling DNA repair^13^. These include positive selection of the gene *Apex1* (involved in the control of ER) in *Heterocephalus glaber*^14^, amplification of *Fbxo31* (involved in the DNA damage response (DDR)) in the genome of *Myotis brandtii*^15^, and increased expression of *Rpa2* (promotes DNA repair) along with a unique amino acids change in the MMS19 (encoded by *Mms19*) NER protein in *Balaena mysticetus*^16^. The bowhead whale also has unique mutations in the ER gene *Ercc1* and the PCNA gene, both involved in DNA replication and RAD6-dependent post-replicative DNA repair^17^.

At present, only limited data are available on the effects of overexpression of the DNA repair genes on longevity, and no studies have addressed the impact of stress, an important longevity factor, on these effects. In mice, a positive effect on longevity is observed with overexpression of human enzyme hMTH1, which eliminates oxidized purine^18^ and deacetylase *Sirt6*^19^. Overexpression of SIRT6 promotes DSB repair by activating PARP1 and facilitating the recruitment of Rad51^20^ and NBS1^21^ to DNA lesions. In the nervous system of *D. melanogaster*, overexpression of DDR genes *GADD45* and *PARP1* has a lifespan extending effect^22^,^23^. Additionally, introduction of 1–2 extra copies of the gene *mei-41* (homologous to *ATR* gene in mammals) into the genome of *D. melanogaster* leads to an increase in lifespan compared to wild-type flies^24^. At the same time, overexpression of the gene O^6^-methylguanine-DNA-methyltransferase (*hMGMT*) in the tissues of mice does not result in increased longevity^25^, and widespread ectopic expression of the gene *hPARP1* in mice^26^ leads to a decrease in survival.

The purpose of this study was to determine whether overexpression of genes involved in the control of various DNA repair pathways would result in an increased lifespan and stress resistance in *D. melanogaster*. We studied the effects of overexpression of genes encoding for enzymes coordinating the recognition of DNA damage (homologs of *HUS1, CHK2*), NER and BER (homologs of *XPF, XPC* and *AP-endonuclease-1*), and DSB repair (homologs of *BRCA2, XRCC3, KU80* and *WRNexo*) on lifespan and resistance to stress factors (hyperthermia, oxidative stress and starvation). Most of the UAS-bearing transgenic flies for overexpression of DNA repair genes, including *UAS-Ku80* (*Ku80* homologue), *UAS-mei-9* (*XPF* homologue), *UAS-mus210* (*XPС* homologue), *UAS-Rrp1* (orthologue of *APE1*), and *UAS-WRNexo* (orthologue of WRN 3′–5′ exonuclease domain), were produced for the first time for this study. Because a variety of types of age-related accumulation of DNA damage exist, we used DNA repair genes that control most of the known mechanisms of DNA repair (Fig. 1).

## Results

We investigated the effects of overexpression of DNA repair genes on *D. melanogaster* lifespan and stress resistance. To activate the expression of DNA repair genes, we used the GAL4-UAS binary regulatory system^27^. We crossed transgenic flies (holding extra copies of the gene of interest under control of the *UAS* promoter) with flies with GAL4 drivers. We then assessed lifespan in the offspring.

Because somatic mutations accumulate with age in a tissue-specific manner^5^, we activated the overexpression of DNA repair genes both globally and tissue-specifically. Tissue-specific overexpression was activated in the nervous system. This system was selected for several reasons. First, nerve cells are chronically exposed to oxidative stress and thus vulnerable to accumulating DNA damage^28^. Secondly, aging of the brain leads to onset and progression of neurological diseases, which accelerate and aggravate the aging process^29^. And finally, many transgenes have been identified that can increase lifespan when over-expressed in neurons^30^. Thus, the nervous system may be considered a key target of anti-aging interventions.

We used drivers constitutively active throughout all stages of the life cycle (*1407-GAL4* and *da-GAL4*) and conditionally activated (by RU486 feeding) in the adult stage only (*Elav-GS* and *Act5C-GS*). These drivers activate the gene overexpression in a tissue-specific manner in the nervous system (*1407-GAL4* and *Elav-GS*) or ubiquitously in all tissues (*da-GAL4* and *Act5C-GS*)^31^,^32^.

### Lifespan effects

#### Constitutive/ubiquitous

**Figure 2:**
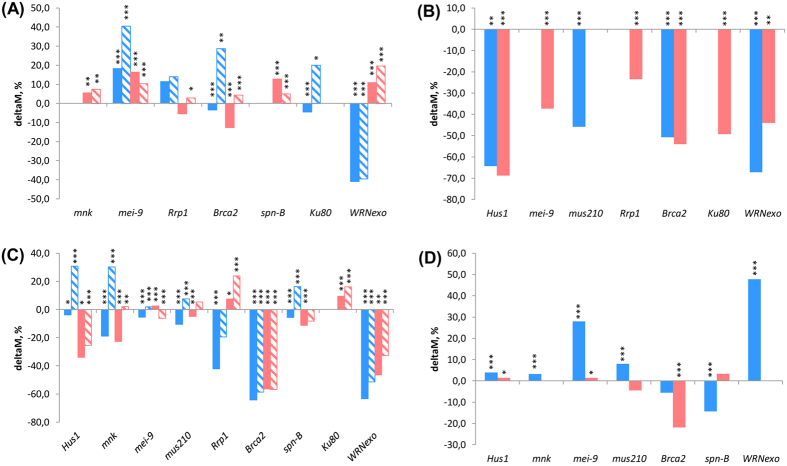
The impact of overexpression of DNA repair genes driven by *da-GAL4* (A), *Act5C-GS* (B), *1407-GAL4* (C), and *Elav-GS* (D) on life span of males (blue) and females (red). Overexpression was driven by *da-GAL4* (**A**), *Act5C-GS* (**B**), *1407-GAL4* (**C**), and *Elav-GS* (**D**). The columns with solid colors show differences with *da-GAL4>w* and hatching columns with *w>UAS*. The results of 1–4 replicates are combined. The Y-axis shows the values of differences in median lifespan in %. *p < 0.05, **p < 0.01, ***p < 0.001, Mantel-Cox test.

The expression level of DNA repair genes under the control of a ubiquitous constitutive driver *da-GAL4* increased by 1.2–3.5 fold (Supplementary Fig. S1). This resulted in an increase of the median lifespan in males by 7–40% (with overexpression of *mnk, mei-9,* and *spn-B*) and in females by 10–30% (with overexpression of *mnk, mei-9, spn-B,* and *WRNexo;* Fig. 2A and Supplementary Table S2). Notably, the positive effect of the *da-GAL4*-driven overexpression of *Brca2* and *Ku80* (males only) was only observed in comparison with short-lived *UAS* control flies. Also, the large increase in expression of *WRNexo* (8.7 fold) in males (Supplementary Fig. S1) actually decreased median lifespan by 40% (Fig. 2A and Supplementary Table S2).

#### Conditional/Ubiquitous

Under the control of the conditional ubiquitous driver *Act5C-GS*, the expression level of *Hus1, mei-9, mus210, Rrp1, Brca2, Ku80,* and *WRNexo* increased by 1.5–15.6 fold in males and 1.2–5.7 fold in females (Supplementary Fig. S1). Conditional ubiquitous activation of DNA repair genes resulted in a reduction of the median lifespan in male and female flies by 49–72% (Fig. 2B and Supplementary Table S2).

#### Constitutive/neurospecific

Under the control of constitutive neurospecific driver *1407-GAL4,* the expression level of *Hus1, mnk, mus210, Rrp1, spn-B, Brca2,* and *Ku80* increased by 1.5–5.5 fold in males and 1.5–4 fold in females (Supplementary Fig. S1). The relative expression level of *WRNexo* and *mei-9* increased 36.5–50.5 fold in males and 11.5–30.5 fold in females, respectively (Supplementary Fig. S1). This resulted in a reduction of the median lifespan of males by 4–64%. In females, the overexpression of genes *spn-B, Brca2, Hus1, mnk, mus210* and *WRNexo* reduced lifespan by 5–56%, while overexpression of *Ku80* and *Rrp1* led to an increase of 3–9% (Fig. 2C and Supplementary Table S2). Notably, the positive effect of the 1407-GAL4-driven overexpression of *Hus1, mnk, mus210, mei-9* and *spn-B* (in males) was observed only in the background of short-lived *UAS* controls.

#### Conditional/neurospecific

The relative expression levels of DNA repair genes under the control of conditional neurospecific driver *Elav-GS* increased 1.3–8.2 fold in the nervous tissue of imago males and 1.4–7 fold in female flies (Supplementary Fig. S1). Meanwhile, the overexpression of *WRNexo* in females increased 16.7 fold. The *Elav-GS*-driven overexpression resulted in increased median lifespan in males overexpressing *Hus1* (4%), *mnk* (3% ), *mei-9* (28%), *mus210* (8%), *WRNexo* (48%) and slightly in females overexpressing genes *Hus1* (1.5%) and *mei-9* (1.5%) (Fig. 2D and Supplementary Table S2). The median lifespan was reduced in males with conditional gene overexpression of *spn-B* (14%) and *Ku80* (2%), as well as in females with the overexpressed gene *Brca2* (21%; Fig. 2D and Supplementary Table S2).

Thus, the most significant lifespan-extending effect was found in flies with constitutive ubiquitous overexpression of *mnk, spn-B, and WRNexo* (in females) and *mei-9* (in both sexes), genes under control of *da-GAL4* driver.

### Stress resistance

Stress can have a variety of detrimental effects on an organism. To reveal the role of overexpression of DNA repair genes in organismal stress resistance, we analyzed fly survival under constant conditions of hyperthermia, oxidative stress (paraquat), and starvation. Treatment of flies with paraquat^33^ and high temperature may cause somatic mutations to accumulate^5^, while nutrient deprivation may impair DNA repair processes^34^. Stress resistance results are presented in Table 1.

#### Hyperthermia

Consitutive overexpression of DNA repair genes had an overall positive effect on resistance to hyperthermia. Resistance to hyperthermia increased in males with constitutive ubiquitous overexpression of all DNA repair genes (*da-GAL4* driver) and in flies of both sexes with constitutive neurospecific overexpression of all genes except *spn-B* in females (*1407-GAL4* driver; Supplementary Fig. S2). Conditional overexpression, on the other hand, had mixed results. The conditional ubiquitous overexpression (*Act5C-GS* driver) of *WRNexo* in both sexes and *Hus1* in females led to an increase in resistance to hyperthermia (Supplementary Fig. S2), but the conditional ubiquitous overexpression of *Hus1, mei-9, mus210, Brca2* in males and *Rrp1* and *Ku80* in females led to a decrease (Supplementary Fig. S2). Similarly, the conditional neurospecific overexpression of *mnk* and *WRNexo* in males and *mei-9* and *Hus1* in females (*Elav-GS* driver) led to an increase in hyperthermia resistance (Supplementary Fig. S2), but the conditional neurospecific overexpression of *mus210, Brca2*, and *spn-B* in both sexes and *Hus1* in males led to a decrease (Supplementary Fig. S2).

Thus, constitutive overexpression of DNA repair genes under control of *da-GAL4* and *1407-GAL4* drivers, respectively, whether throughout the body or confined to the nervous system, demonstrated a predominantly positive effect on thermotolerance. Conversely, conditional ubiquitous or neurospecific overexpression under control of *Act5C-GS* and *Elav-GS* drivers, respectively, either increased or decreased resistance to higher temperature depending on the gene studied, with decreased resistance predominating.

#### Oxidative stress

Resistance to oxidative stress decreased after activation of overexpression of all DNA repair genes in all experimental conditions (Supplementary Fig. S2), except males with constitutive ubiquitous overexpression (*da-GAL4* driver) of *mei-9, Rrp1*, and *Ku80* and females with conditional ubiquitous overexpression (*Act5C-GS* driver) of *Hus1* or conditional neurospecific expression (*Elav-GS* driver) of *mei-9* and *Brca2* (Supplementary Fig. S2).

Thus, constitutive ubiquitous overexpression under control of *da-GAL4* in males had the most positive effect on resistance to oxidative stress, but the overall effect of the DNA repair genes’ overexpression, with a few exceptions, was decreased oxidative stress resistance.

#### Starvation

Ubiquitous overexpression of DNA repair genes had both positive and negative effects on starvation resistance whether constitutive or conditional, depending on the genes overexpressed. For example, the constitutive ubiquitous overexpression of *Rrp1* in males (*da-GAL4* driver) increased resistance to starvation (Supplementary Fig. S2); however, resistance to starvation decreased after activation of constitutive ubiquitous overexpression of *Brca2* and *WRNexo* in males and *Rrp1* and *WRNexo* in females (Supplementary Fig. S2). Likewise, the conditional ubiquitous expression of *Brca2* and *WRNexo* in males and *Ku80* in females (*Act5C-GS* driver) increased resistance to starvation (Supplementary Fig. S2), but the conditional ubiquitous expression of *mei-9* and *mus210* in males and *Hus1* and *Brca2* in females decreased resistance to starvation (Supplementary Fig. S2). Neurospecific overexpression of DNA repair genes, whether constitutive or conditional, had a negative effect on starvation resistance. Constitutive neurospecific overexpression (*1407-GAL4* driver) decreased resistance to starvation after activation of all DNA repair genes in males and in females (Supplementary Fig. S2), and conditional neurospecific expression (*Elav-GS* driver) of *spn-B* and *Brca2* in males and *mei-9* in females also decreased resistance to starvation (Supplementary Fig. S2).

Thus, resistance to starvation was most positively affected by overexpression of DNA repair genes with conditional ubiquitous expression, under the control of *Act5C-GS* driver; however, both positive and negative effects of ubiquitous expression, whether constitutive or conditional, occurred and were dependent on gene and sex. Any effects of neurospecific expression on starvation resistance were negative.

Generally, these data demonstrate that the positive effects of the overexpression of DNA repair genes on resistance to different stressors are more evident in males. The constitutive ubiquitous overexpression of *mei-9, Rrp1, Brca2, Ku80*, and *WRNexo* genes, under control of *da-GAL4* driver, are the most beneficial.

## Discussion

Here, we have shown that increasing the expression level of DNA repair genes in *Drosophila melanogaster* has both positive and negative effects on lifespan and stress resistance depending on the type of GAL4 driver used, the genes overexpressed, and, in some cases, sex of the organism (Table 1).

### Increased lifespan with constitutive ubiquitous and conditional neuronal overexpression

The constitutive ubiquitous overexpression of most DNA repair genes tested resulted in increased lifespan. This effect was seen in all experimental conditions, except when *Ku80* and *WRNexo* were overexpressed in males and *Brca2* in females. Conditional neuronal activation of expression only at the adult stage also increased lifespan, with the exception of *Brca2* when overexpressed in females, and *spn-B* and *Ku80* when overexpressed in males.

### Reduced lifespan with conditional ubiquitous or constitutive neuronal overexpression

Conversely, when overexpression of DNA repair genes occurred throughout the body but was limited to adulthood, lifespan was reduced. At first sight, this may suggest that RU486 (myfepristone) negatively impacts longevity. However, according to a recent study by Landis et al., RU486 does not affect the lifespan of males and virgin females and actually may increase the lifespan of mating females up to 68%^35^. In addition, RU486 increases lifespan observed using the *Elav-GS* driver. Thus, while RU486 remains a possible factor, the available evidence does not support this. We also observed shorter lifespan in flies with constitutive overexpression of DNA repair genes in the nervous system in all experimental conditions, with the exception of *Rrp1* and *Ku80* in females. One possible explanation for this is that ectopic overexpression of DNA repair genes under control of *Act5C-GS* and *1407-GAL4* drivers may disturb cell energy metabolism and intracellular signaling pathways, decreasing organismal viability.

Thus, overexpression of DNA repair genes throughout development leads to opposite effects on lifespan when compared to adult-specific overexpression, and the direction of this dichotomy depends on whether the overexpression was ubiquitous or limited to the nervous system. It is difficult to explain these effects on the basis of the available experimental or published data, but it is possible that transcriptome analysis carried out at different stages of development could be informative.

In addition, there were opposing effects on the two sexes, depending on driver. Increased lifespan driven by *da-GAL4* was more pronounced in females, but the same driven by *Elav-GS* was observed specifically in males, with the exception of *Hus1*. Sex-specific effects of transgenes that can increase lifespan when overexpressed are well known^35^.

### Identification of candidate genes for future studies of life extension via DNA repair

The ambiguous effect of constitutive versus adult-specific overexpression may be also related to the different functions of genes and different levels of their activity. In accordance with our data, the most positive effects on lifespan were observed in flies with constitutive ubiquitous overexpression of *mnk* (in both sexes), *mei-9, spn-B*, and *WRNexo* (in females) and in flies with conditional neurospecific overexpression of *Hus1* and *mei-9* (in both sexes), *mnk, mus210*, and *WRNexo* (in males). These genes are involved in various DNA damage recognition and repair mechanisms.

*Drosophila mei-9* is essential for several DNA repair and recombination pathways, including NER, interstrand crosslink repair, and meiotic recombination^36^. In the mammalian 9-1-1 complex, Hus1 forms a DNA damage sensor clamp^37^. In *Drosophila,* the *Hus1* homologue plays a critical role in the regulation of the S-phase meiotic DNA damage checkpoint and DSB repair during meiotic recombination^37^,^38^. *Mnk* (also known as *Chk2*) is involved in regulating the activity of the DNA damage sensors Ku70 and Ku80^39^; the overexpression of *Ku80* is characterized by a positive effect on the lifespan. *Mus210* (also known as *XPC*) may act as a general sensor of damaged DNA^40^. These data are supported by reports of a positive correlation between the activity of DNA damage-sensing enzymes such as Ku80^12^ and PARP1 and longevity of different species of animals^11^ and reports that increased gene expression of DNA damage sensors *mei-41* (throughout the body) and *PARP1* (in the nervous system) also leads to increased longevity in *Drosophila melanogaster*^23^,^24^. Thus, the ability of enzymatic systems to recognize DNA damage may influence longevity.

Moderate expression of *WRNexo* in female flies increased longevity, while high expression in males substantially reduced lifespan. The reasons for this remain unclear, but several possibilities exist. First, WRNexo is known to be an orthologue of human WRN 3′–5′ exonuclease domain^41^ and it is known that excess nuclease activity, e.g. XPF (ortholog of mei-9)^42^, leads to DNA damage and genomic instability. Secondly, because the DNA repair process is ATP-dependent, high levels of *WRN* may lead to the depletion of energy and cell death. *Drosophila* WRNexo shows conservation of structural motifs and catalytic residues with human protein, but lacks a helicase domain^43^. WRNexo is required for response to replicative stress, restraining of mitotic DNA recombination, and maintenance of genome stability^43^,^44^. In *Drosophila* cells lacking *WRNexo*, collapsed replication forks persist and promote Holliday junction formation and HR^43^. Additionally, WRNexo degrades SSD, duplex DNA substrates, and bubble structures, but has no effect on blunt ended DNA duplexes^45^. Taken together, these findings point to its possible involvement in DNA excision repair and DSB repair.

Overexpression of *spn-B* and *Brca2*, both globally and in the nervous system, had a predominantly negative effect on lifespan. This may be related to the fact that *spn-B* and *Brca2* both control the processes of HR. These processes are of paramount importance during mitosis and meiosis^46^ but do not play a significant role in the post-mitotic cells of the adult organism. The gene *spn-B* is required for the progression of the meiotic cell cycle^47^. Rad51-related proteins spn-B and spn-D physically interact and promote HR during meiotic prophase with accompanied suppression of the NHEJ repair pathway^48^. Mutations in DNA repair genes such as *spn-B* lead to persistence of DSBs in the germline, which activates an ATR-Chk2-dependent checkpoint^49^. *Drosophila Brca2* loss-of-function sufficiently decreases HR repair with compensatory error-prone repair predominates. Brca2 provides both mitotic and meiotic DSB repair and the transduction of the meiotic recombination checkpoint signals^46^. Brca2 physically interacts with spn-A (Rad51 homologue) and recruits to DNA damage. Its activity is processed DSB repair by gene conversion^50^.

It is also important that DNA repair is carried out by large multienzyme complexes, as imbalance of one component may lead to its inefficiency. For example, supplementation of Rad51, Rad51C, Rad52, and NBS1 proteins in human fibroblasts, either individually or in combination, did not rescue the senescence-related decline of homologous recombination without overexpression of deacetilase SIRT6^20^. In this regard, it would be useful to assess the effects on lifespan of overexpression of several proteins from a single DNA repair pathway.

### DNA repair and the prevention of neurodegeneration in aging

Evidence suggests that that the nervous system plays a critical role in longevity and the aging process^51^. The results of the current study lend support for this view by demonstrating that lifespan in *Drosophila* can be increased by overexpression of DNA repair genes in the adult nervous system alone (under the control of the neurospecific driver *Elav-GS*). We have also previously demonstrated that *Elav-GS-*specific overexpression of DNA repair genes such as *PARP1* and *D-GADD45* in the *Drosophila* nervous system is sufficient to increase the lifespan of the whole organism^22^,^23^. While the mechanisms underlying these lifespan effects are not immediately apparent, one possibility is that neurospecific overexpression of DNA repair genes may prevent the development of age-dependent neurodegeneration. In line with this is the reverse scenario, in which DNA damage causes neurodegeneration. Indeed, the loss of heterochromatin and subsequent accumulation of DNA damage in the *Drosophila* brain have been shown to promote neurodegeneration^52^. Moreover, other experiments involving overexpression of *Ku70* and *D-GADD45* confirm that DNA repair genes are important for maintaining the normal functions of neurons and the prevention of age-related neurodegeneration^51^,^53^.

Alternatively, it is also possible that the longer lifespan observed using the *Elav-GS* driver could include effects of mifepristone^35^, or, since the process of DNA repair is also closely linked with aging-related mechanisms such as cell cycle regulation, apoptosis, autophagy, and IGF-1 signaling^54^,^55^, lifespan may have been extended via alterations of aging-related cell signaling pathways. Finally, it is important to consider that nervous system-specific overexpression of DNA repair genes actually decreased life span when the overexpression was constitutive instead of conditional to adulthood. Thus, the lifespan effects are influenced by driver or stage of development.

### Stress Resistance

Lifespan and stress resistance are interrelated and DNA repair can affect both^56^. The three stressors selected for this study (hyperthermia, paraquat, and starvation) each have specific detrimental effects. Hyperthermia causes nuclear protein aggregation and stalling of DNA replication forks and leads to the induction of DNA damage, including DSB^57^. Paraquat induces reactive oxygen species-mediated DNA damage^58^. Starvation may impair DNA repair processes, as many steps in DNA repair are ATP dependent^34^.

While overexpression of DNA repair genes in the absence of stressors had a more pronounced effect in females, the beneficial effects of overexpression of these genes on resistance to stress was more pronounced in males. The effects of overexpression of DNA repair genes on different types of stress resistance were varied. Constitutive ubiquitous overexpression of the majority of the studied DNA repair genes led not only to increased lifespan in males, but also improved resistance to hyperthermia and oxidative stress (Table 1), whereas conditional ubiquitous overexpression under the control of the *Act5C-GS* driver in imagoes resulted in reduced lifespan but increases in resistance to hyperthermia, oxidative stress and starvation in male and female flies (Table 1). Consitutive neurospecific overexpression of DNA repair genes, under the control of *1407-GAL4* driver, increased the resistance to hyperthermia, but reduced lifespan and resistance to oxidative stress and starvation. The correlation between stress resistance and lifespan were most closely correlated in the cases of conditional neurospecific overexpression of the *spn-B* (reduction), *mnk, Rrp1* and *WRNexo* (increase) in males, and *Brca2* (reduction), *Hus1* and *mei-9* (increase) in females, under the control of driver *Elav-GS*.

Different stress factors may induce DNA damage via the generation of free radicals^59^. The observed increases in stress resistance may reflect elevated efficiency of DNA repair. However, the involvement of alternative mechanisms affecting such stress-resistance mechanisms as cell cycle regulation, apoptosis, autophagy, and IGF-1 signaling^54^,^55^ are also possible. Thus, our results are consistent with stress resistance being necessary, but not sufficient, for longevity.

## Conclusions

Aging is a complex process that is far from being fully understood. Of the many factors that contribute to aging and the multiple changes on many levels that take place, one in need of further study at this time is the role of DNA repair. Because DNA damage does accumulate with age and appears to be associated with some of the detrimental aspects of aging, including neurodegeneration, boosting DNA repair mechanisms may be one approach to intervention. Here, we investigated the potential life-extending effects of increasing the expression of genes known to be involved in DNA repair in *Drosophila*. We compared the overexpression of these genes throughout the body versus in the nervous system alone and throughout the lifespan versus in adulthood alone. We also included three known stressors. We found both positive and negative effects on lifespan, with many important variables, including gene, sex, stress exposure, extent of overexpression, and type of *GAL4* driver used, which determined developmental stage and distribution of overexpression in the body. The most pronounced effects of overexpression on lifespan occurred with *Hus1, mnk, mei-9, mus210, spn-B*, and *WRNexo.* which control the processes of DNA damage recognition and repair. Lifespan and stress resistance were interrelated, moreso in males than females, in that increased lifespan was associated with increased resistance to hyperthermia and oxidative stress, while decreased lifespan was associated with decreased resistance to all three stressors tested. Aging research is still in need of basic studies to address a wide variety of unanswered questions. This study presents a valuable set of preliminary data on the role of DNA repair in aging and points to a promising set of DNA repair genes and experimental conditions to pursue in greater detail in future studies that incorporate both transcription-level and protein-level effects on a wider variety of lifespan- and aging-related parameters.

## Materials and Methods

### Drosophila strains

In order to match the genetic background of UAS and GAL4 strains utilized in this study, flies all were backcrossed into *w*^*1118*^ (#3605, Bloomington *Drosophila* Stock Center) background for 6–8 times.

### UAS strains

*Hus1* (genotype: *w*^*1118*^*, UAS-Hus1*)—Carries an additional copy of gene *Hus1* under the *UAS* promoter’s control on chromosome 2. Hus1 is a protein from the PCNA-like complex 9-1-1 that is required for the activation of an S phase checkpoint^60^ and DSB repair during meiotic recombination^38^. Kindly provided by Dr. Schupbach (Princeton University, Princeton, USA).

*mnk* (genotype: *w*^*1118*^*, UAS-mnk*)—Carries an additional copy of an ortholog of the mammalian DNA damage sensor gene *chk2* under the control of promoter *UAS* on chromosome 2^61^. Kindly provided by Dr. Schupbach (Princeton University, Princeton, USA).

*mei-9* (genotype: *w*^*1118*^*, UAS-mei-9*)—Carries an additional copy of ortholog of the mammalian excision DNA repair gene *XPF* under the control of promoter *UAS* on chromosome 2^62^. Ordered from GenetiVision (GenetiVision Houston, USA), with authorship transfer.

*mus210* (genotype: *w*^*1118*^*, UAS-mus210*)—Carries an additional copy of ortholog of the mammalian excision DNA repair gene *XPC* under the control of promoter *UAS* on chromosome 3^63^. Ordered from GenetiVision (GenetiVision Houston, USA), with authorship transfer.

*Rrp1,* (genotype: *w*^*1118*^*, UAS-Rrp1*)—Carries an additional copy of ortholog of the mammalian excision DNA repair gene *APE1* under the control of promoter *UAS* on chromosome 2^64^. Ordered from GenetiVision (GenetiVision Houston, USA), with authorship transfer.

*Brca2* (genotype: *w*^*1118*^*, UAS-Brca2*,—Carries an additional copy of *Drosophila* ortholog of mammalian *Brca2* gene under the *UAS* promoter’s control on chromosome 2. Brca2 is involved in DSB repair^46^,^50^,^65^. Kindly provided by Dr. Schupbach (Princeton University, Princeton, USA).

*spn-B* (genotype: *w*^*1118*^*, UAS-spn-B*)—Carries an additional copy of an ortholog of the mammalian DSB repair gene *XRCC3* under the control of promoter *UAS* on chromosome 2^61^. Kindly provided by Dr. Schupbach (Princeton University, Princeton, USA).

*Ku80* (genotype: *w*^*1118*^*, UAS-Ku80*)—Carries an additional copy of gene *Ku80* under the control of *UAS* promoter on chromosome 3. Ku80 is involved in the DSB repair by NHEJ^66^. Ordered from GenetiVision (GenetiVision Houston, USA), with authorship transfer.

*WRNexo* (genotype: *w*^*1118*^*, UAS-WRNexo*)—Carries an additional copy of gene *WRNexo* under the control of promoter *UAS* on chromosome 3. *WRNexo* is the orthologue of human WRN 3′–5′ exonuclease domain involved in DSB repair^41^. *Drosophila* WRNexo shows conservation of structural motifs and catalytic residues with human protein, but lacks a helicase domain^43^. Ordered from GenetiVision (GenetiVision Houston, USA), with authorship transfer.

### Driver GAL4 strains

*da-GAL4* (genotype: *w1118; P{da-GAL4.w-}3*)—Expresses GAL4 ubiquitously and strongly under the control of *daughterless*^67^. This driver expresses throughout development and in most adult tissues^31^. Kindly provided by Dr. Seroude, (Queen’s University, Kingston, Canada).

*Act5c-GS* (genotype: *P{Act5C(-FRT) GAL4.Switch.PR}3/TM6B, Tb1*)—Expresses mifepristone-inducible GAL4 in all cells. Provided by *Drosophila* Stock Center (#9431, Bloomington, USA).

*1407-GAL4* (genotype: w*; P{GawB}insc^*Mz1407*^)—Driver line containing *GAL4* selectively expressed in nervous system cells throughout the life cycle: during embryonic^68^ and larval^69^ stages and imagoes^70^. Provided by *Drosophila* Stock Center (#8751, Bloomington, USA).

*Elav-GS* (genotype: *P{ELAV- GeneSwitch}*)—Expresses mifepristone-inducible GAL4 in nervous system cells^71^. Kindly provided by Dr. Keshishian (Yale University, New Haven, USA).

### Activation of overexpression

The GAL4-UAS system were used to activate the expression of DNA repair genes^27^. We assessed the lifespan in the offspring obtained by mating the transgenic flies with extra copies of the studied gene under *UAS* promoter and flies with GAL4 drivers. We used constitutively active (*1407-GAL4* and *da-GAL4*) and conditional (*Elav-GS* and *Act5C-GS*) drivers of GAL4 that activate the gene overexpression in neurons and throughout the body, respectively.

To activate the overexpression under the control of conditional drivers, adult flies were fed on yeast paste containing mifepristone (RU486, Sigma, USA) at a concentration of 200 μM^32^. Mifepristone was administered in the diet of flies throughout their lifespan. Control animals were fed with yeast paste without mifepristone. To prepare 100 ml of the paste, 50 g of dried yeast and 60 ml of water were used. To exclude the probability of absorption of the active substance by live yeast, the paste was pre-boiled in a water bath for 30 minutes. Five days after placing the flies on the yeast paste containing mifepristone, their stress-resistance and the relative expression levels of genes of interest were evaluated.

### Quantitative Real Time PCR (qRT-PCR)

To confirm overexpression of studied genes in the whole body or nervous system ten imagoes or 50 heads were used in every variant of the experiment. Gene expression levels were analyzed in flies at the age of 2–5 days after imago hatching, separately for males and females. Experiments were performed in 3–4 replicates. Whole flies or heads were homogenized with the Silent Crusher-S homogenizer (Heidolph, Germany) in TRIzol Reagent (Invitrogen, USA). RNA was separated using BCP (Invitrogen, USA), in accordance with the manufacturer’s protocol. To test that RNA samples were DNA-free, control PCR experiments without the reverse transcription step were performed with primers for the *β-Tubulin* gene. Reverse transcription was performed using an Oligo(dT)_20_ primer (Invitrogen, USA) and SuperScript III Reverse Transcriptase (Invitrogen, USA), according to manufacturer’s instructions.

Quantitative real-time PCR (qRT-PCR) assays were performed using SYBRGreen PCR Master Mix (Applied Biosystems, USA). The list of primers is presented in Table S3. All reactions were performed using a CFX96 real-time PCR detection system (Bio-Rad Laboratories, USA). The thermal cycle conditions were: initial denaturation step at 95 °С for 10 min, followed by 50 cycles of 95 °С for 15 s (denaturation), 60 °С for 30 s (annealing) and 60 °С for 30 s (elongation). Expression levels were normalized against the housekeeping gene *β-Tubulin.* All target genes and *β-Tubulin* were amplified in separate PCR tubes. Four measurements were performed for each version of the experiment.

### Lifespan assay

We used flies with statistically significant overexpression for the lifespan assay. Control and experimental flies were collected during 24 h after imago hatching, and divided into males and non-virgin females and maintained in a constant climate chamber Binder KBF720-ICH, 720 l-(Binder, Germany) on a yeast medium at 25 °C and 60% humidity in a 12:12 h light-dark cycle. Thirty flies of the same sex and age were maintained in a *Drosophila* vial. Five vials were used in each experiment (a total of 150 males and 150 females). Experiments were performed in several replicates. Flies were transferred to a fresh medium twice a week. Lifespan was analyzed daily, separately for males and females. The median lifespan and the age of 90% mortality were calculated. The data are presented in the form of histograms reflecting the percentage of changes in median lifespan between experimental and control variants.

### Estimation of stress resistance

We used flies with statistically significant overexpression for the stress-resistance estimation. Evaluation of stress-resistance (to hyperthermia, oxidative stress and starvation) was performed in the flies at the age of 5 days. To induce hyperthermia, the flies were kept at 35 °C. To trigger oxidative stress, the flies were kept on filter paper moistened with 5% sucrose solution with the addition of paraquat at 20 mM concentration. Starved flies were kept on filter paper moistened with distilled water. Flies with overexpression of DNA repair genes and without overexpression lived under stress conditions until the whole experimental group died. The survival was evaluated every 24 hours. The results obtained are presented in the form of histograms reflecting the percentage of dead flies after 24–96 hours.

### Statistics

To compare the statistical differences in median lifespan between control and experimental groups, the Mantel-Cox test was used^72^. A Wang-Allison test was used to estimate the differences in the age of 90% mortality^73^. To assess the statistical significance of differences in resistance to stress factors, the Fisher’s exact test was used^74^. Relative levels of expression were calculated using 2^-ΔΔСt^ method^75^. *ΔΔCt* was calculated according to equation *ΔΔCt = ΔCt (experiment) − ΔCt (control),* where *ΔCt = Ct (target gene) − Ct (β-Tubulin).* Statistical significance of expression differences was estimated using Mann-Whitney U-test^76^. Statistical analyses of the data were performed using STATISTICA software, version 6.1 (StatSoft, USA), R, version 2.15.1, and OASIS: Online Application for the Survival Analysis of Lifespan Assays^74^.

## Additional Information

**How to cite this article**: Shaposhnikov, M. *et al.* Lifespan and Stress Resistance in *Drosophila* with Overexpressed DNA Repair Genes. *Sci. Rep.* **5**, 15299; doi: 10.1038/srep15299 (2015).

## Supplementary Material

Supplementary Information

## Figures and Tables

**Figure 1:**
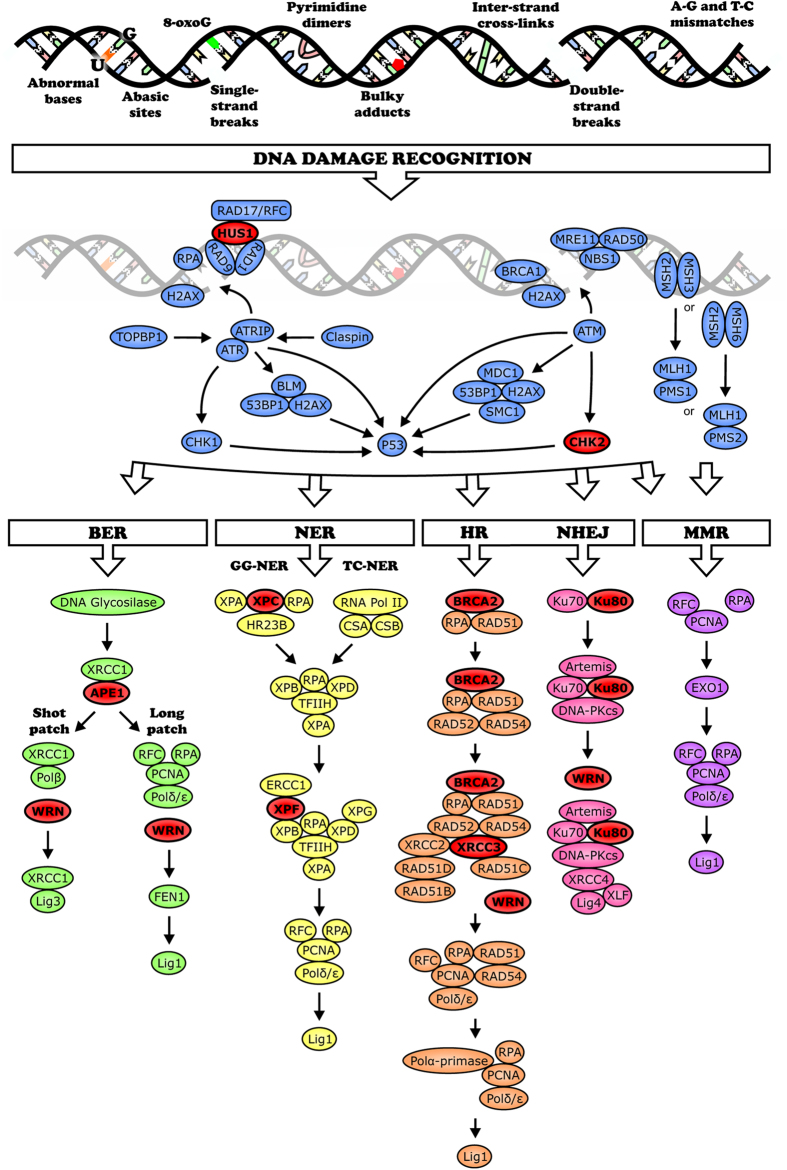
The types of DNA damage seen with the age-related increase of DNA damage level and the associated repair mechanisms in human and mammalian cells. The proteins whose *Drosophila* homologic genes were overexpressed in the present study are highlighted in red.

**Table 1:** Effect of overexpression of DNA repair genes on median lifespan and stress resistance.

Gene, DNA Repair Process, and Gene Function	Sex	Driver															
		*da-GAL4*				*Act5C-GS*				*1407-GAL4*				*Elav-GS*			
		ML	HT	OS	ST	ML	HT	OS	ST	ML	HT	OS	ST	ML	HT	OS	ST
*Hus1*, DDR, damage response protein	M					↓	↓	↓	—	↓	↑	—	↓	↑	↓	—	—
	F					↓	↑	↑	↓	↓	↑	—	—	↑	↑	↓	—
*mnk*, DDR, chk2 protein kinase	M									↓	↑	↓	↓	↑	↑	—	—
	F	↑	—	↓	—					↓	—	↓	—				
*mei-9*, NER, DNA repair endonuclease	M	↑	↑	↑	—	—	↓	↓	↓	↓	↑	↓	↓	↑	—	—	—
	F	↑	—	—	—	↓	—	—	—	↑	↑	↓	—	↑	↑	↑	↓
*mus210*, NER, DNA damage recognition	M					↓	↓	—	↓	↓	↑	↓	↓	↑	↓	—	—
	F									↓	↑	↓	—	—	↓	↓	—
*Rrp1*, BER, apurinic endonuclease and DNA 3′ exonuclease	M	—	↑	↑	↑					↓	↑	—	↓				
	F	—	—	—	↓	↓	↓	↓	—	↑	↑	↓	—				
*Brca2*, DSBR, binds the single strand DNA	M	↓	↑	—	↓	↓	↓	↓	↑	↓	↑	—	↓	—	↓	↓	↓
	F	↓	—	—	—	↓	↓	—	↓	↓	—	↓	—	↓	↓	↑	—
*spn-B*, DSBR, binds the single strand DNA	M									↓	↑	—	↓	↓	↓	—	↓
	F	↑	—	—	↑					↓	↓	↓	↓	—	↓	↓	—
*Ku80*, DSBR, synapsis of DNA ends	M	↓	↑	↑	—												
	F					↓	↓	—	↑	↑	↑	↓	—				
*WRNexo*, DSBR, 3′->5′ exonuclease	M	↓	↑	—	↓	↓	↑	—	↑	↓	↑	↓	↓	↑	↑	—	—
	F	↑	—	—	↓	↓	↑	—	—	↓	↑	↓	↓	—	—	—	—

ML – median lifespan; HT – hyperthermia; OS – oxidative stress; ST – Starvation; M – males; F – females; ↑ – increase; ↓ – decrease; — – no statistically significant effects; empty cell—data are not analyzed due to the lack of statistically significant differences in the level of gene expression.
