# Supplementary material for: Lifespan and Stress Resistance in Drosophila with Overexpressed DNA Repair Genes
Source: Sci Rep. 2015 Oct 19;5:15299. doi: 10.1038/srep15299 (PMC4609912; doi:10.1038/srep15299)
Supplement: Supplementary Information [file srep15299-s1.pdf]

Lifespan and Stress Resistance in *Drosophila* with Overexpressed DNA Repair Genes

Mikhail Shaposhnikov, Ekaterina Proshkina, Lyubov Shilova, Alex Zhavoronkov and Alexey Moskalev*

Supplementary Tables

Supplementary Table S1. Age-dependent changes in the efficiency of DNA repair

DNA repair mechanism	Repair efficiency	References
Base excision repair	↓	[1], [2]
Nucleotide excision repair	↓	[3]
Mismatch repair	↓	[4]
Single-strand break repair	↓	[5], [6]
Homologous recombination	↑	[7]
Single-strand annealing	↓	[7]
Non-homologous end joining	↓	[8]

Supplementary Table S2. Influence of DNA repair genes overexpression on the lifespan

Genotype, +/-RU486	Sex	90%	P(90%)	M	P(M)	n(rep)	n
			***da-GAL4* driver**				
da-GAL4>w	M	61		44		4	564
w>UAS-mei-9	M	58		37		1	161
da-GAL4>UAS-mei-9	M	70	3E-05[a], 0.0001[c]	52	1.2E-11[a], 0[c]	1	158
w>UAS-Rrp1	M	59		43		3	438
da-GAL4>UAS-Rrp1	M	63	0.668[a], 0.668[d]	49	0.122[a], 0.526[d]	3	449
w>UAS-Brca2	M	52		33		1	157
da-GAL4>UAS-Brca2	M	55	0.0002[a], 2E-05[e]	42.5	6.86E-06[a], 0.0039[e]	1	154
w>UAS-Ku80	M	51		35		2	297
da-GAL4>UAS-Ku80	M	51	0[a], 0.2490[f]	42	1.96E-10[a], 0.0185[f]	2	266
w>UAS-WRNexo	M	57		43		1	153
da-GAL4>UAS-WRNexo	M	54	0.829[a], 0.0004[h]	26	9.81E-07[a], 0.0003[h]	1	45
da-GAL4>w	F	67		55		4	555
w>UAS-mnk	F	66		54		1	141
da-GAL4>UAS-mnk	F	70	0.009[a], 0.723[b]	58	0.002[a], 0.0071[b]	1	207
w>UAS-mei-9	F	64		58		1	150
da-GAL4>UAS-mei-9	F	74	0[a], 0[c]	64	0[a], 0[c]	1	178
w>UAS-Rrp1	F	66		50.5		3	386
da-GAL4>UAS-Rrp1	F	68	0.578[a], 0.0703[d]	52	0.055[a], 0.0269[d]	3	423
w>UAS-Brca2	F	53		46		1	122
da-GAL4>UAS-Brca2	F	61	0.039[a], 1E-05[e]	48	3.12E-08[a], 0.0002[e]	1	147
w>UAS-spn-B	F	61		59		1	157
da-GAL4>UAS-spn-B	F	72	0.0003[a], 0[g]	62	1.64E-07[a], 5E-11[g]	1	159
w>UAS-WRNexo	F	64		51		1	146
da-GAL4>UAS-WRNexo	F	77	0[a], 0[h]	61	6.15E-07[a], 0[h]	1	123
			***Act5C-GS* driver**				
UAS-Hus1/Act5C-GS, -RU486	M	60		42		2	402
UAS-Hus1/Act5C-GS, +RU486	M	24	0[a]	15	0[a]	2	433
UAS-mei-9/Act5C-GS, -RU486	M	39		56		2	385
UAS-mei-9/Act5C-GS, +RU486	M	40	0.6825[c]	56	0.446[c]	2	411
UAS-mus210/Act5C-GS, -RU486	M	39		59		2	431
UAS-mus210/Act5C-GS, +RU486	M	16	0[d]	32	0[d]	2	396
UAS-Brca2/Act5C-GS, -RU486	M	40		63		2	499
UAS-Brca2/Act5C-GS, +RU486	M	12	0[f]	31	0[f]	2	473
UAS-WRNexo/Act5C-GS, -RU486	M	43		61		2	422
UAS-WRNexo/Act5C-GS, +RU486	M	14	0[i]	20	0[i]	2	353
***UAS-Hus1/Act5C-GS,* -RU486**	F	64		48		2	409
UAS-Hus1/Act5C-GS, +RU486	F	49	3E-05[a]	15	0[a]	2	415
UAS-mei-9/Act5C-GS, -RU486	F	39		59		2	463
UAS-mei-9/Act5C-GS, +RU486	F	18	0[c]	37	0[c]	2	439

UAS-Rrp1/Act5C-GS, -RU486	F	44		64		2	365
UAS-Rrp1/Act5C-GS, +RU486	F	13	0[e]	49	0[e]	2	522
UAS-Brca2/Act5C-GS, -RU486	F	49		63		2	552
UAS-Brca2/Act5C-GS, +RU486	F	16	0[f]	29	0[f]	2	449
UAS-Ku80/Act5C-GS, -RU486	F	58		71		2	349
UAS-Ku80/Act5C-GS, +RU486	F	24	0[h]	36	0[h]	2	588
UAS-WRNexo/Act5C-GS, -RU486	F	46		59		2	410
UAS-WRNexo/Act5C-GS, +RU486	F	19	0[i]	33	0[i]	2	296
***1407-GAL4* driver**							
***1407-GAL4>w*[a]**	M	74		53		2	189
***1407-GAL4> w*[b]**	M	61		48		2	283
***1407-GAL4> w*[c]**	M	72		55		2	245
***1407-GAL4> w*[d]**	M	63		49		2	175
***1407-GAL4>w*[e]**	M	67		50		2	145
w>UAS-Hus1	M	50		39		2	191
1407-GAL4>UAS-Hus1	M	63	0.005[a], 0[f]	51	0.023[a], 0[f]	2	246
w>UAS-mnk	M	53		33		2	215
1407-GAL4>UAS-mnk	M	64	0.061[a], 0[g]	43	2.71E-05[a], 1.15E-09[g]	2	182
w>UAS-mei-9	M	53		51		2	280
1407-GAL4>UAS-mei-9	M	58	0[c], 0[h]	52	0[c], 1.31E-09[h]	2	227
w>UAS-mus210	M	46		40		2	268
1407-GAL4>UAS-mus210	M	55	0.0002[b], 0[i]	43	7.36E-08[b], 0[i]	2	250
w>UAS-Rrp1	M	51		36		1	159
1407-GAL4>UAS-Rrp1	M	50	0[e], 0.4331[j]	29	0[e], 0.0984[j]	2	160
w>UAS-Brca2	M	57		46		2	202
1407-GAL4>UAS-Brca2	M	48	0[a], 0.0007[k]	19	0[a], 0[k]	2	146
w>UAS-spn-B	M	55		43		2	209
1407-GAL4>UAS-spn-B	M	69	0.093[a], 0[l]	50	0.13[a], 4E-12[l]	2	257
w>UAS-WRNexo	M	61		37		2	254
1407-GAL4>UAS-WRNexo	M	32	0[d], 0[n]	18	0[d], 0[n]	2	197
***1407-GAL4>w*[a]**	F	80		62		2	243
***1407-GAL4> w*[b]**	F	76		63		2	241
***1407-GAL4> w*[c]**	F	73		58.5		2	158
***1407-GAL4> w*[d]**	F	82		69		2	280
***1407-GAL4>w*[e]**	F	64		53		2	147
w>UAS-Hus1	F	78		55		2	174
1407-GAL4>UAS-Hus1	F	49	0[a], 0[f]	41	0[a], 0[f]	2	227
w>UAS-mnk	F	62		47		2	226
1407-GAL4>UAS-mnk	F	57	0[a], 0[g]	48	0[a], 0.0014[g]	2	132
w>UAS-mei-9	F	81		64		2	245
1407-GAL4>UAS-mei-9	F	70	0.7756[c], 0[h]	60	0.849[c], 0[h]	2	264
w>UAS-mus210	F	71		57		2	248

1407-GAL4>UAS-mus210	F	74	1E-04[b], 0.0043[i]	60	0.0004[b], 0.237[i]	2	228
w>UAS-Rrp1	F	63		46		1	145
1407-GAL4>UAS-Rrp1	F	65	0.0323[e], 0.0605[j]	57	0.0012[e], 3.12E-06[j]	2	147
w>UAS- Brca2	F	74		62.5		2	260
1407-GAL4>UAS-Brca2	F	53	1E-05[a], 0.0155[k]	27	0[a], 0[k]	2	125
w>UAS- spn-B	F	73		60		2	257
1407-GAL4>UAS-spn-B	F	72	0[a], 0.481[l]	55	9.19E-09[a], 0.17[l]	2	231
w>UAS-Ku80	F	65		50		1	141
1407-GAL4>UAS-Ku80	F	75	2E-05[e], 0[m]	58	1.37E-09[e], 1.8E-08[m]	2	122
w>UAS-WRNexo	F	74		55		2	258
1407-GAL4>UAS-WRNexo	F	50	0[d], 0[n]	37	0[d], 0[n]	2	245
***Elav-GS* driver**							
Elav-GS>UAS-Hus1, -RU486	M	76		64		2	324
Elav-GS>UAS-Hus1, +RU486	M	81	0.0354[a]	66.5	2.41E-07[a]	2	339
Elav-GS>UAS-mnk, -RU486	M	71		62		2	342
Elav-GS>UAS-mnk, +RU486	M	82	0[b]	64	3E-12[b]	2	371
Elav-GS>UAS-mei-9, -RU486	M	65		50		1	108
Elav-GS>UAS-mei-9, +RU486	M	72	2E-05[c]	64	0[c]	1	125
Elav-GS>UAS-mus210, -RU486	M	64		50		1	140
Elav-GS>UAS-mus210, +RU486	M	68	5E-05[d]	54	8.25E-05[d]	1	123
Elav-GS>UAS-Brca2, -RU486	M	65		54		2	337
Elav-GS>UAS-Brca2, +RU486	M	67	0.4472[e]	51	0.196[f]	2	270
Elav-GS>UAS-spn-B, -RU486	M	64		56		2	270
Elav-GS>UAS-spn-B, +RU486	M	61	1E-05[f]	48	4.5E-11[g]	2	322
Elav-GS>UAS-WRNexo, -RU486	M	64		44		1	137
Elav-GS>UAS-WRNexo, +RU486	M	72	3E-04[g]	65	0[i]	1	140
Elav-GS>UAS-Hus1, -RU486	F	84		68		2	328
Elav-GS>UAS-Hus1, +RU486	F	88	0.0096[a]	69	0.0012[a]	2	338
Elav-GS>UAS-mei-9, -RU486	F	77		70		1	149
Elav-GS>UAS-mei-9, +RU486	F	78	0.012[c]	71	0.0131[c]	1	112
Elav-GS>UAS-mus210, -RU486	F	78		68		1	162
Elav-GS>UAS-mus210, +RU486	F	78	0.0031[d]	65	0.187[d]	1	150
Elav-GS>UAS-Brca2, -RU486	F	75		64		2	360
Elav-GS>UAS-Brca2, +RU486	F	71	1E-05[e]	50	0[f]	2	379
Elav-GS>UAS-spn-B, -RU486	F	76		59		2	281
Elav-GS>UAS-spn-B, +RU486	F	74	0.003[f]	61	0.867[g]	2	278
Elav-GS>UAS-WRNexo, -RU486	F	74		64		1	142
Elav-GS>UAS-WRNexo, +RU486	F	73	0.079[g]	64	0.288[i]	1	116

-RU486, no mifepristone (no overexpression); +RU486, mifepristone (overexpression); M, Male; F, Female; 90%, 90th percentile; P(90), P-value (Wang-Allison test); P(M), P-value (Mantel-Cox test): NS, not significant; n(rep), number of experiments; n, number of flies.

Controls for *da-GAL4>UAS*: [a]*da-GAL4>w* and [b]*UAS-mnk*; [c]*UAS-mei-9*; [d]*UAS-Rrp1*; [e]*UAS-Brca2*; [f]*UAS-Ku80*; [g]*UAS-spn-B*; [h]*UAS-WRNexo*.

Controls for *Act5C-GS>UAS*: [a]*Act5C-GS>UAS-Hus1*, -RU486; [b]*Act5C-GS>UAS-mnk*, -RU486; [c]*Act5C-GS>UAS-mei-9*, -RU486; [d]*Act5C-GS>UAS-mus210*, -RU486; [e]*Act5C-GS>UAS-Rrp1*, -RU486; [f]*Act5C-GS>UAS-Brca2*, -RU486; [g]*Act5C-GS>UAS-spn-B*, -RU486; [h]*Act5C-GS>UAS-Ku80*, -RU486; [i]*Act5C-GS>UAS-WRNexo*, -RU486.

Controls for *1407-GAL4>UAS*: [a]*1407-GAL4>*w^{a}; [b]*1407-GAL4>*w^{b}; [c]*1407-GAL4>* w^{c}; [d]*1407-GAL4>* w^{d}; [e]*1407-GAL4>*w^{e}; [f]*w>UAS-Hus1*; [g]*w>UAS-mnk*; [h]*w>UAS-mei-9*; [i]*w>UAS-mus210*; [j]*w>UAS-Rrp1*; [k]*w>UAS- Brca2*; [l]*w>UAS-spn-B*; [m]*w>UAS-Ku80*; [n]*w>UAS-WRNexo*.

Controls for *Elav-GS>UAS*: [a]*Elav-GS>UAS-Hus1*, -RU486; [b]*Elav-GS>UAS-mnk*, -RU486; [c]*Elav-GS>UAS-mei-9*, -RU486; [d]*Elav-GS>UAS-mus210*, -RU486; [e]*Elav-GS>UAS-Brca2*, -RU486; [f]*Elav-GS>UAS-spn-B*, -RU486; [g]*Elav-GS>UAS-WRNexo*, -RU486.

Supplementary Table S3. Primers for quantitative RT-PCR

Gene	Forward	Reverse
β-Tubulin	5'-GCAACTCCACTGCCATCC-3'	5'-CCTGCTCCTCCTCGAACT-3'
Hus1	5'-TGATGCAGGATCCGCTGTACATGA-3'	5'-TCCTCAGCTGTAATTCCTGCCCAA-3'
mnk	5'-ATGTGCCATGCCGTCAAGTACCTA-3'	5'-TCCTCGTCATTGGTCTCCAGCAAA-3'
mei-9	5'-TCCTCAAGGCCTACAGCGATTC-3'	5'-TCCAGATAAACGCGCTCTCTTTC-3'
mus210	5'-AGAAGACGGTGCATTTGAGATTGC-3'	5'-CCTCGCAAACAATGAAGCCATCG-3'
Rrp1	5'-AGGATGGTCTGCAGTTGATTGAC-3'	5'-GTTTGCGCACTTGGTTTCCTG-3'
Brca2	5'-TCGTCGCCGTGGAGGATCTTATTT-3'	5'-TCTGCGTATGTTGGAGACGAGCAA-3'
spn-B	5'-AGATTGCTGCAGATGAGCAAAGCC -3'	5'-TTTATAACGCACGCCAGGAGAGGT-3'
Ku80	5'-GAGCTTCAGAATGTCGCAACTACC-3'	5'-GGAAAGTCGTTGAAATCGAAGAGC -3'
WRNexo	5'-TGGTGGCCCTTATCAATCATCCC-3'	5'-GTGCCAGCTTTCGGAAATCGTTC-3'

Supplementary Figures

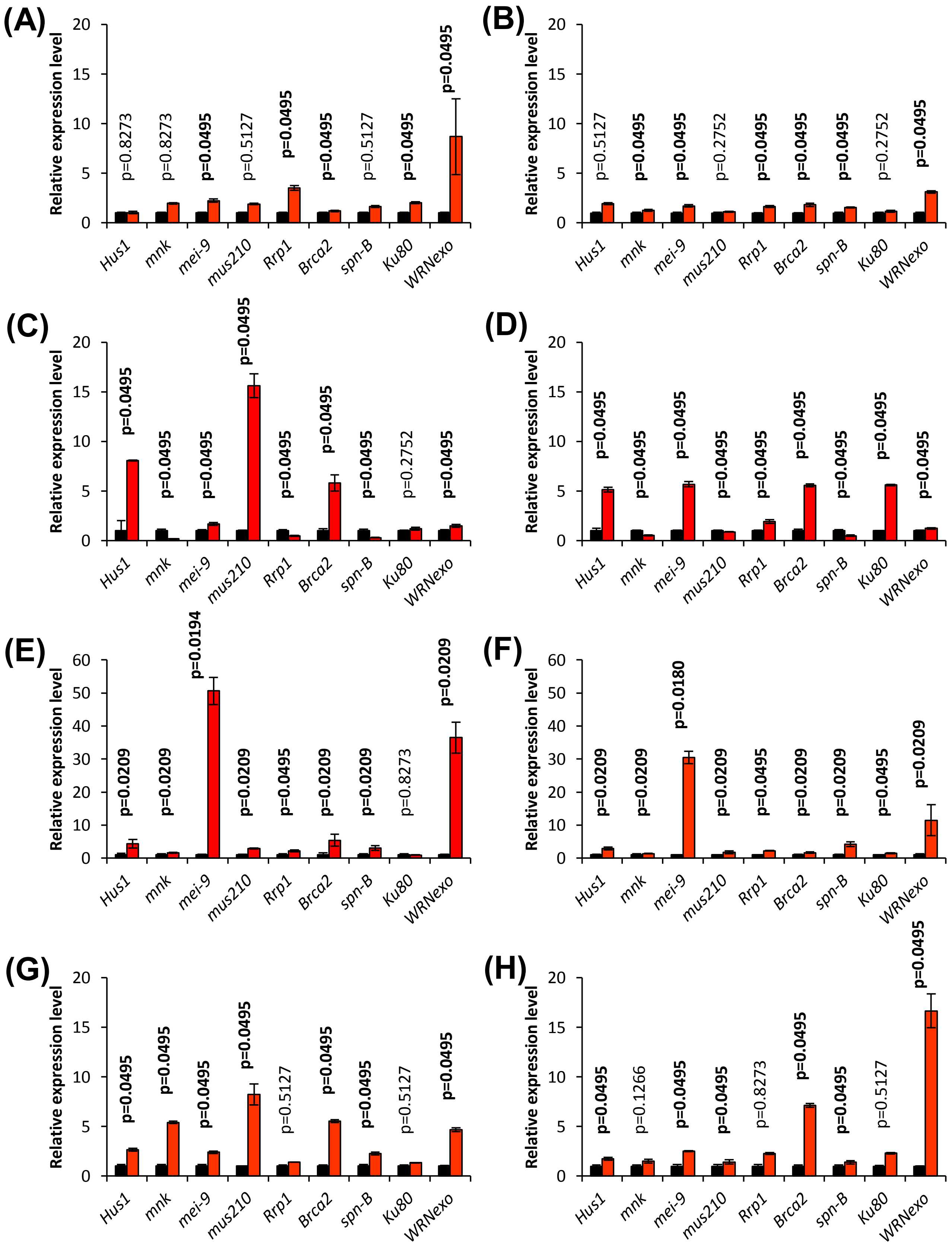

Supplementary Figure S1. The relative level of expression of DNA repair genes in control flies (black) and flies with overexpression (red).

Overexpression under control of *da-GAL4* (A, males and B, females), *Act5C-GS* (C, males and D, females), *1407-GAL4* (E, males and F, females), and *Elav-GS* (G, males and H, females). P-values were calculated by Mann-Whitney U-test. The error bars represent standard error of the mean.

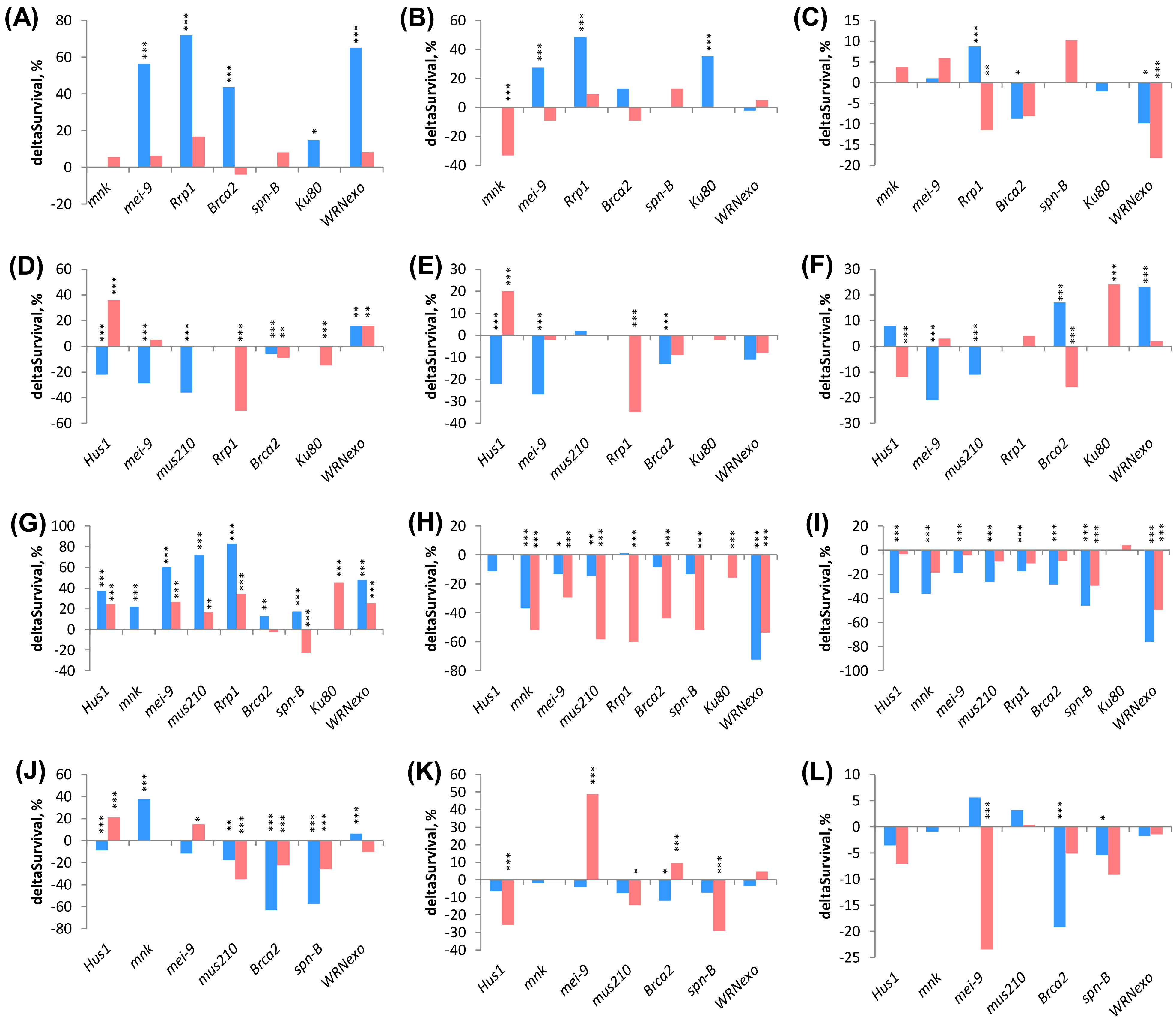

Supplementary Figure S2. The influence of overexpression of DNA repair genes on the stress resistance of males (blue) and females (red). Overexpression under control of *da-GAL4* (A, B, C), *Act5C-GS* (D, E, F), *1407-GAL4* (G, H, I), and *Elav-GS* (J, K, L). Survival after hyperthermia (A, D, G, J), oxidative stress (B, E, H, K), and starvation (C, F, I, L). *p<0.05, **p<0.01, ***p<0.001, Fisher's exact test.

Supplementary References

1 Atamna, H., Cheung, I. & Ames, B. N. A method for detecting abasic sites in living cells: Age-dependent changes in base excision repair. *Proc. Natl. Acad. Sci. USA* **97**, 686-691 (2000).

2 Rao, K. S., Annapurna, V. V., Raji, N. S. & Harikrishna, T. Loss of base excision repair in aging rat neurons and its restoration by DNA polymerase beta. *Mol. Brain Res.* **85**, 251-259 (2000).

3 Vijg, J., Mullaart, E., Lohman, P. H. & Knook, D. L. UV-induced unscheduled DNA synthesis in fibroblasts of aging inbred rats. *Mutat. Res.* **146**, 197-204 (1985).

4 Toyota, M. *et al.* CpG island methylator phenotype in colorectal cancer. *Proc. Natl. Acad. Sci. USA* **96**, 8681-8686 (1999).

5 Newton, R. K., Ducore, J. M. & Sohal, R. S. Effect of age on endogenous DNA single-strand breakage, strand break induction and repair in the adult housefly, *Musca domestica*. *Mutat. Res.* **219**, 113-120 (1989).

6 Singh, N. P., Danner, D. B., Tice, R. R., Brant, L. & Schneider, E. L. DNA damage and repair with age in individual human lymphocytes. *Mutat. Res.* **237**, 123-130 (1990).

7 Preston, C. R., Flores, C. & Engels, W. R. Age-dependent usage of double-strand-break repair pathways. *Curr. Biol.* **16**, 2009-2015, doi:10.1016/j.cub.2006.08.058 (2006).

8 Frasca, D. *et al.* Effect of age on DNA binding of the ku protein in irradiated human peripheral blood mononuclear cells (PBMC). *Exp. Gerontol.* **34**, 645-658 (1999).
